# Supplementary material for: Patterns of Intron Gain and Loss in Fungi
Source: PLoS Biol. 2004 Nov 30;2(12):e422. doi: 10.1371/journal.pbio.0020422 (PMC532390; doi:10.1371/journal.pbio.0020422)
Supplement: Table S1 — Also available at http://genes.mit.edu/NielsenEtAl/. (4.3 MB ZIP). [file pbio.0020422.st001.zip › NielsenEtAl/html/769.html]

AN5746.1.NCU10042.1.MG10607.1.FG01346.1


```
 CLUSTAL W (1.82) Multiple Sequence Alignments - Introns Inserted


Sequence 1: NCU10042.1	438 aa
Sequence 2: MG10607.1	423 aa
Sequence 3: FG01346.1	438 aa
Sequence 4: AN5746.1	438 aa
Alignment Length: 438 aa
Number Identitical Residues: 330 aa
Alignment Score (without introns) 13657


MG10607.1 	MAITKIHARSVYDSRGNPTVEVDVVTDTGLNRAIVPSGASTG1QHEACELRDGDKSKWGG
NCU10042.1	MPISKIHARYVYDSRGNPTVEVDVVTELGLHRAIVPSGASTG1QHEACELRDGDKTKWGG
FG01346.1 	MAIKKVFARSVYDSRGNPTVEVDVVTETGLHRAIVPSGASTG1QHEACELRDGDKSKWNG
AN5746.1  	MPISKIHARSVYDSRGNPTVEVDVVTETGLHRAIVPSGASTG1QHEAHELRDGDKSKWLG
          	*.*.*:.** ****************: **:*********** **** *******:** *

MG10607.1 	KG~VTKAVENVNNIIGPALIKENIDVKDQSKVDEFLIQLDGTPNKTKLGANAILGVSLAV
NCU10042.1	KG~VLKAVQNVNEVIGPALIKENIDVKDQSKVDKFLIDLDGTPNKTKLGANAILGVSLAV
FG01346.1 	KG~VTKAVENVNTVIAPALIEKNLDVKDQSAVDAFLNELDGTTNKTKLGANAILGVSLAV
AN5746.1  	KG1VLTAVKNVNETIGPAVIKENLDVKEQSKIDEFLNKLDGTPNKSNLGANAILGVSLAI
          	** * .**:***  *.**:*::*:***:** :* ** .****.**::************:

MG10607.1 	AKAGAAAK0---------------KPYVLPVPFMNVLNGG2SHAGGRLAFQEFMIVPS2A
NCU10042.1	AKAGAAEK~GVPLYAHISDLAGTKKPYVLPVPFMNVLNGG2SHAGGRLAFQEFMIVPS~A
FG01346.1 	AKAGAAEK0GVPLYAHVSDLAGTKKPYVLPVPFMNVLNGG~SHAGGRLAFQEFMIVPT2D
AN5746.1  	AKAGAAEK~GVPLYAHISDLAGTKKPYVLPVPFQNVLNGG~SHAGGRLAFQEFMIVPD2T
          	****** * . .  :  :. :.:.********* ****** ****************   

MG10607.1 	APSFTEAMRQGAEVYQQLKGLAKKKYGQSAGNVGDEGGVAPDIQSAEEALELITAAIEAA
NCU10042.1	APTFSEALRQGAEVYQILKSLAKKKYGQSAGNVGDEGGVAPDIQNPEEALDLITEAIEKA
FG01346.1 	APTFSEALRQGAEVYQALKGLAKKRYGQSAGNVGDEGGVAPDIQTAEEALELITDAIEQA
AN5746.1  	ASSFSEGLRQGAEVYHKLKALAKKKYGQSAGNVGDEGGVAPDIQTAEEALDLITEAIEQA
          	*.:*:*.:*******: **.****:*******************..****:*** *** *

MG10607.1 	GYTGKMNIAMDVASTEFYKEDEKKYDLDFKNPDSDKSKWITYEELAAMYSDLCKKYPIVS
NCU10042.1	GYTGQVKIAMDVASSEFYKEDVKKYDLDFKNPESDPSKWLTYEELANLYSELCKKYPIVS
FG01346.1 	GYTGQMKIAMDVASSEFYKESEKKYDLDFKNPESDPTKWITYEELANMYSELCKKYPIVS
AN5746.1  	GYTGKIHIAMDVASSEFYKPEEKKYDLDFKNPDSDPSKWLTYEQLADLYKSLAAKYPIVS
          	****:::*******:**** . **********:** :**:***:** :*..*. ******

MG10607.1 	IEDPFAEDDWEAWSYFYKTQDIQIVA~DDLTVTNPLRIKKAIDQKAANALLLKVNQIGTL
NCU10042.1	IEDPFAEDDWEAWSYFYKTQDIQIVA~DDLTVTNPLRIKKAIELKAANALLLKVNQIGTL
FG01346.1 	IEDPFAEDDWEAWSYFSKTQDIQVVG~DDLTVTNPLRIKKAIELKSCNALLLKVNQIGTL
AN5746.1  	IEDPFAEDDWEAWSYFYKTSDFQIVG2DDLTVTNPLRIKKAIELKSCNALLLKVNQIGTL
          	**************** **.*:*:*. ***************: *:.*************

MG10607.1 	TESIQAAKDSFADGWGVMVSHRSGETEDVTIADIAVGLRAGQIKTGAPARSERLAKLNQI
NCU10042.1	TESIQAAKDSYADGWGVMVSHRSGETEDVTIADIVVGIRSGQIKTGAPARSERLAKLNQI
FG01346.1 	TESIQAAKDSYADGWGVMVSHRSGETEDVTIADIAVGLRAGEIKTGAPARSERLAKLNQI
AN5746.1  	TESIQAAKDSYADGWGVMVSHRSGETEDVTIADISVGLRSGQIKTGAPARSERLAKLNQI
          	**********:*********************** **:*:*:******************

MG10607.1 	LRIEEELGSNAVFAGESFRKAINM
NCU10042.1	LRIEEELADNAIFAGEKFRKAVEL
FG01346.1 	LRIEEELGDQAIYPGKDFRKSVNL
AN5746.1  	LRIEEELGENAVYAGQNFRKSVNL
          	*******..:*::.*:.***::::
```
